# Autoimmune encephalitis as a differential diagnosis of schizophreniform psychosis: clinical symptomatology, pathophysiology, diagnostic approach, and therapeutic considerations

**DOI:** 10.1007/s00406-020-01113-2

**Published:** 2020-03-12

**Authors:** Dominique Endres, Frank Leypoldt, Karl Bechter, Alkomiet Hasan, Johann Steiner, Katharina Domschke, Klaus-Peter Wandinger, Peter Falkai, Volker Arolt, Oliver Stich, Sebastian Rauer, Harald Prüss, Ludger Tebartz van Elst

**Affiliations:** 1 Section for Experimental Neuropsychiatry, Department of Psychiatry and Psychotherapy, Medical Center - University of Freiburg, Faculty of Medicine, University of Freiburg, Freiburg, Germany; 2 Department of Psychiatry and Psychotherapy, Medical Center - University of Freiburg, Faculty of Medicine, University of Freiburg, Freiburg, Germany; 3 Department of Neurology, Christian-Albrechts-University Kiel, Kiel, Germany; 4 Neuroimmunology Section, Institute of Clinical Chemistry, University Hospital Schleswig-Holstein Kiel/Lübeck, Lübeck, Germany; 5 Department of Psychiatry and Psychotherapy 2, Ulm University, Bezirkskrankenhaus Günzburg, Günzburg, Germany; 6 Department of Psychiatry and Psychotherapy, University Hospital, Ludwig-Maximilians-University, Munich, Germany; 7 Department of Psychiatry, Psychotherapy and Psychosomatics of the University Augsburg, Bezirkskrankenhaus Augsburg, University of Augsburg, Augsburg, Germany; 8 Department of Psychiatry and Psychotherapy, University of Magdeburg, Magdeburg, Germany; 9 Department of Psychiatry, University of Münster, Münster, Germany; 10 Department of Neurology, Medical Center - University of Freiburg, Faculty of Medicine, University of Freiburg, Freiburg, Germany; 11 Medical Care Center, Neurology, Konstanz, Germany; 12 Department of Neurology and Experimental Neurology, Charité-Universitätsmedizin Berlin, Berlin, Germany; 13 German Center for Neurodegenerative Diseases (DZNE) Berlin, Berlin, Germany

**Keywords:** Schizophrenia, Psychosis, Antibody, Autoimmune encephalitis, Autoimmune psychosis, CSF

## Abstract

Primary schizophreniform psychoses are thought to be caused by complex gene–environment interactions. Secondary forms are based on a clearly identifiable organic cause, in terms of either an etiological or a relevant pathogenetic factor. The secondary or “symptomatic” forms of psychosis have reentered the focus stimulated by the discovery of autoantibody (Ab)-associated autoimmune encephalitides (AEs), such as anti-NMDA-R encephalitis, which can at least initially mimic variants of primary psychosis. These newly described secondary, immune-mediated schizophreniform psychoses typically present with the acute onset of polymorphic psychotic symptoms. Over the course of the disease, other neurological phenomena, such as epileptic seizures, movement disorders, or reduced levels of consciousness, usually arise. Typical clinical signs for AEs are the acute onset of paranoid hallucinatory symptoms, atypical polymorphic presentation, psychotic episodes in the context of previous AE, and additional neurological and medical symptoms such as catatonia, seizure, dyskinesia, and autonomic instability. Predominant psychotic courses of AEs have also been described casuistically. The term autoimmune psychosis (AP) was recently suggested for these patients. Paraclinical alterations that can be observed in patients with AE/AP are inflammatory cerebrospinal fluid (CSF) pathologies, focal or generalized electroencephalographic slowing or epileptic activity, and/or suspicious “encephalitic” imaging findings. The antibody analyses in these patients include the testing of the most frequently found Abs against cell surface antigens (NMDA-R, CASPR2, LGI1, AMPA-R, GABA_B_-R), intracellular antigens (Hu, Ri, Yo, CV2/CRMP5, Ma2 [Ta], amphiphysin, GAD65), thyroid antigens (TG, TPO), and antinuclear Abs (ANA). Less frequent antineuronal Abs (e.g., against DPPX, GABA_A_-R, glycine-R, IgLON5) can be investigated in the second step when first step screening is negative and/or some specific clinical factors prevail. Beyond, tissue-based assays on brain slices of rodents may detect previously unknown antineuronal Abs in some cases. The detection of clinical and/or paraclinical pathologies (e.g., pleocytosis in CSF) in combination with antineuronal Abs and the exclusion of alternative causes may lead to the diagnosis of AE/AP and enable more causal therapeutic immunomodulatory opportunities.

## Background

Schizophrenia and other psychotic disorders are severe and frequent conditions characterized by delusions, hallucinations, disorganization, formal thinking changes, catatonia, and different negative symptoms typically occurring for the first-time during adolescence and early adulthood [65]. Primary schizophreniform psychoses are caused by a complex interaction between multiple genes and environmental factors [81]. Large, genome-wide studies have identified over 100 distinct gene sites that contribute to the relative risk of psychotic symptoms [73]. Secondary forms are based on clearly identifiable causes in the sense of etiology or according to recognizable pathogenesis [45, 81]. Such secondary forms can be linked to autoantibody (Ab)-associated autoimmune processes such as anti-*N*-Methyl-d-aspartate receptor [NMDA-R] encephalitis [44]. In 2007, the field of autoimmune encephalitis (AE) was redefined with the first description of anti-NMDA-R encephalitis [16, 18, 19]. Since then, a large number of other antineuronal Abs against cell surface antigens and their associated syndromes have been identified [15, 17, 31, 89, 90]. Because these syndromes can be accompanied by polymorphic psychotic symptoms, immunological concepts of schizophreniform psychoses have gained considerable attention since [1, 6, 15, 22, 36, 67, 68, 76, 77, 78, 84]. In a German case series of 100 patients with different forms of AEs with Abs against antineuronal antigens, over half of the patients (60%) presented with psychotic symptoms [36]. In most cases that are positive for antineuronal Abs, patients develop clear neurological symptoms in the course of the disease, such as dystonic movement disorders or epileptic seizures [31, 36, 51]. For AE with predominant psychotic symptoms, the term “autoimmune psychosis” (AP) was recently suggested [21, 61, 67]. The changing nomenclature for autoimmune neuropsychiatric phenomena is summarized in Box 1.

Box 1: Different nomenclature [6, 22] *Encephalopathy:* Traditionally, this term has been used mainly for persistent brain damage. The term has also been used when secondary brain damage was assumed, but the exact mechanism of the disease remained unclear (e.g., hepatic or epileptic encephalopathy). Because antineuronal autoantibodies (Abs) can now be detected, cases of encephalopathy not previously recognized as neuroinflammatory can comply with the criteria of autoimmune encephalitis.

*Limbic encephalitis (LE):* LE has developed in the context of paraneoplastic encephalitis, which has been known for some time and has undergone a change in meaning in the discourse of the last decade. Originally, the term described a clinical focal point syndrome. Currently, the term is mainly used syndromally, as a description of a clinical syndrome [31].

*Autoimmune encephalitis (AE):* The term has largely established itself as an umbrella term for Ab-associated immune-mediated neuropsychiatric syndromes. It is also used to describe Ab-negative, probable AE. In an international consensus paper from global experts in the field of neurology and neuroimmunology, they have suggested a clinical approach to the diagnosis of autoimmune encephalitis [31].

*Autoimmune psychosis (AP):* AP describes a syndrome with predominant psychoses and a probable autoimmune pathophysiology [21, 61, 67]. Initially, a distinction among the following groups was suggested: (1) psychoses with detection of classical antineuronal Abs, (2) psychoses associated with systemic inflammatory and autoimmune diseases, and (3) Ab-negative AP [61]. The authors of a recent expert consensus paper defined criteria for a possible, probable, and definite AP [67].

### Rationale

The awareness of the fact that psychotic syndromes may have autoimmune, Ab-associated causes opens up a new field in psychiatry for a small but probable relevant subgroup of patients. For clinicians, this raises the question as to how far the diagnostic workup and immunomodulating therapy attempts should be advanced in individual cases. This article investigates this question by illustrating constellations in which extended organic diagnostic procedures, especially Ab analyses, should be carried out.

## Clinical symptomatology

### The syndrome of possible autoimmune encephalitis

In a current consensus article, experts in the field of neurology and neuroimmunology described the syndrome diagnosis of a possible AE. Accordingly, an autoimmune etiology should be considered if the following criteria are present:

1. Subacute onset (less than 3 months) of deficits in working memory, altered mental state (changes in consciousness, changes in personality, or lethargy) or psychiatric (e.g., psychotic) symptoms.
2. One of the following findings:
  New focal neurological symptoms.
  New epileptic seizures.
  Magnetic resonance imaging (MRI) signs of “encephalitis” (temporal FLAIR hyperintensities, multifocal demyelinating or inflammatory lesions).
  Cerebrospinal fluid (CSF) pleocytosis (> 5 per mm^3^).
3. Exclusion of other causes (see Table 4; [31]).

### Established neuropsychiatric syndromes

From a clinical perspective, different established Ab-associated neuropsychiatric syndromes with generally mixed psychiatric and neurological symptoms can be identified (Table 1). In particular, limbic and anti-NMDA-R encephalitis are established central nervous system (CNS) syndromes that can go along with psychotic syndromes [31]. Various Ab-associated immunological systemic diseases, such as the prototype of neuropsychiatric systemic lupus erythematosus (NP-SLE), but also antiphospholipid syndrome, Sjögren’s syndrome, scleroderma, or (ANCA associated) vasculitis may also be associated with psychotic syndromes [63].

**Table 1:** Main neuropsychiatric autoimmune encephalitides associated with psychotic symptoms (adapted from [31]; other references: [3, 5, 7, 10, 14, 18, 22, 38, 40, 43, 46, 48, 80, 86, 91]

	Limbic encephalitis	Anti-NMDA-R encephalitis	Hashimoto encephalopathy	Neuropsychiatric SLE
Age and gender	Mostly elderly patients, but in all ages possible	Especially in girls/young women and children	More common among women; average age 52 years (large range)	Most common in young/middle-aged women
Clinical symptoms and para-clinical findings	1. Subacute onset of working memory deficits, epileptic seizures, or psychiatric symptoms indicating involvement of the limbic system 2. Temporal MRI or FDG-PET pathologies 3. One of the following findings: CSF pleocytosis Temporal EEG pathologies 4. Exclusion of other causes	1. Subacute onset with at least four of the following symptoms: Behavioral or cognitive deficits Speech dysfunction Epileptic seizures Movement disorders, dyskinesia, or rigidity Disturbances of consciousness Autonomic dysfunction or central hypoventilation 2. One of the following findings: EEG changes (incl. extreme delta brush) CSF pleocytosis or oligoclonal bands 3. Exclusion of other causes	1. Encephalopathy with hallucinations, myoclonus, epileptic seizures or stroke-like episodes 2. Subclinical or mild thyroid dysfunction (often hypothyroidism) 3. Normal MRI or nonspecific changes 4. Elevated thyroid Abs in serum 5. No evidence of currently established antineuronal Abs in CSF or serum (incl. “screening” using tissue-based assays) 6. Exclusion of other causes	1. Malar rash 2. Discoid rash 3. Photosensitivity 4. Oral ulcers 5. Non-erosive arthritis 6. Pleuritis/pericarditis 7. Kidney involvement 8. Epileptic seizures or psychosis 9. Hematological involvement, (hemolytic anemia, leukopenia, lymphopenia, thrombopenia) 10. Immunological markers (anti-ds-DNA, Anti-Sm, antiphospholipid Abs) 11 ANA detection
Diagnostic criteria	All four criteria must be fulfilled for diagnosis. If one of criteria 1–3 is not met, the diagnosis can be made only if currently established antineuronal Abs are detected	If all three criteria are met, a syndrome diagnosis can be made. Only three groups of symptoms are required after the detection of a teratoma. The diagnosis is confirmed by Ab detection; in cases of Ab detection, one symptom (under heading 1) is sufficient for diagnosis	All six criteria must be fulfilled (the authors recommend speaking of Hashimoto encephalopathy only when improvement of treatment with steroids or other immunosuppressive procedures was documented → according to the idea of steroid-responsive encephalopathy)	Four criteria (at least one clinical and one immunological criterion) must be fulfilled
EEG	Mostly temporal or frontal epileptic activity and rhythmic delta/theta activity in the EEG	Delta slowing, dysrhythmias, partial epileptic activity/beta-delta complexes, special pattern: the specific finding of “extreme delta brush”	Frequent EEG pathologies with slowing or less often epileptic activity	EEG alterations in approx. 80%, often diffuse theta or delta slowing or epileptic activity
Imaging	Mostly uni- or bilateral mesiotemporal T2/FLAIR hyperintensities; in 10–20% of cases, the MRI remains inconspicuous (in such cases, an FDG-PET might help to objectify the mesiotemporal pathology)	MRI mostly normal, abnormalities only in 33%! T2/FLAIR hyperintensities in the hippocampi, cerebellar, or cerebral cortex, frontobasal, in the insular cortex, in the basal ganglia, and in the brain stem were described	In about half of the patients MRI pathologies, mostly non-specific white matter lesions	MRI changes in 30-75%, mostly T2w hyperintense lesions in the subcortical and deep white matter and around the lateral ventricles, as well as atrophy and cerebral infarction
CSF/serum	Often mild to moderate pleocytosis (i.e., 6–100 cells per mm^3^; in 60–80%, but only in 41% of cases with anti-LGI1 Abs); OCBs in approx. 50% of cases	Moderate pleocytosis, increased total protein concentration, and OCBs (CSF abnormalities in approx. 79%).	Mostly increased anti-TPO and anti-TG Abs (in 69%); less often, isolated increased anti-TPO/TG Abs; often, increased protein concentrations in CSF (82%), and slight CSF pleocytosis in 20%	Increased ANA titers, anti-dsDNA Abs/anti-Sm/anti-rib. P/anti-nucleosome Abs; increased antiphospholipid Abs; CSF pleocytosis in approx. 30%, increased total protein in approx. half of the patients, and OCBs in one-third of those affected

*Abs* antibodies, *CSF* cerebrospinal fluid, *EEG* electroencephalography, *MRI* magnetic resonance imaging, *OCBs* oligoclonal bands

**Fig. 1:**
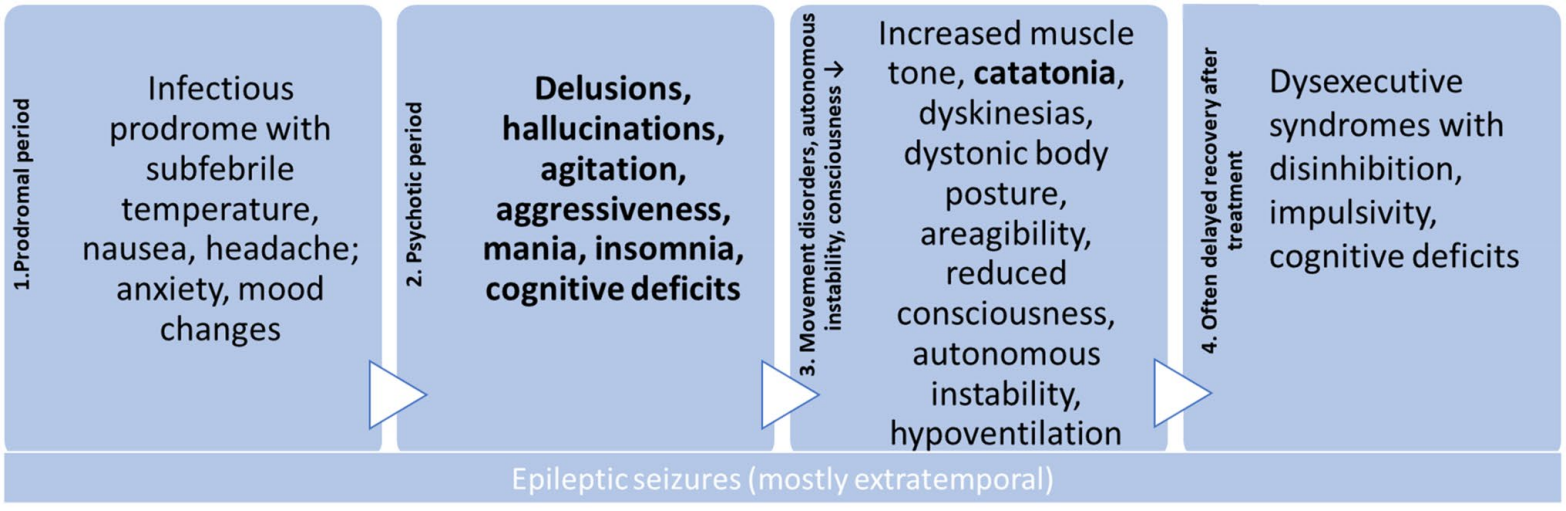
Typical course of anti-NMDA-R encephalitis [14, 15, 18, 22, 52, 70]
- *Hashimoto’s encephalopathy/steroid*-*responsive encephalopathy associated with autoimmune thyroiditis (SREAT):* This is a nosologically unclear, probably etiologically heterogeneous syndromatic diagnosis based on the detection of antibodies against specific thyroid antigens (TPO, TG), non-specific paraclinical findings [e.g., blood–brain barrier (BBB) dysfunction in CSF, electroencephalography (EEG) slowing, MRI white matter lesions, after exclusion of antineuronal Abs in serum and CSF (including tissue-based assay)], and steroid responsiveness [22, 48]. Most authors argue that the thyroid Abs have no functional relevance, are rather indicators of an increased autoimmune susceptibility and that, therefore, this diagnosis will decrease with the further discovery of new, specific antineuronal Abs. In line with these observations, a recent study indicates that the current criteria (see Table 1) do not allow a prediction of steroid responsiveness [57]. Better additional clinical, laboratory or instrumental-based diagnostic parameters as predictors of steroid response need to be explored; the criteria of Hashimoto’s encephalopathy must, therefore, be viewed critically [57].
- *Neuropsychiatric SLE (NP*-*SLE):* The clinical picture of NP-SLE is usually a mixed neurological and psychiatric presentation, with systemic signs often providing decisive diagnostic indications. However, rare cases may present primarily with a classical schizophreniform phenotype [54]. The American College of Rheumatology (ACR) criteria are well established (Table 1), newer classification criteria such as the Systemic Lupus Collaborating Clinics (SLICC) criteria take laboratory findings more into account (https://www.rheumatology.org/Practice-Quality/Clinical-Support/Criteria/ACR-Endorsed-Criteria; [66]).

- *Limbic encephalitis (LE):* LE is characterized by the subacute development of deficits in working memory, paranoid symptoms, hallucinations, irritability, affective symptoms including emotional instability, and epileptic seizures with leading temporal semiology [68]. LE is often associated with specific Abs against cell surface antigens (e.g., LGI-1, GABA_B_-R, and AMPA-R) or intracellular antigens (e.g., GAD65, Hu, and Ma2 [31, 52]).
- *Anti-NMDA-R encephalitis:* This is the most common form of AE, and case series with > 500 patients are published [86]. Tumor association depends on age and gender: in children, tumor association is rare. By contrast, 58% of women from 18 to 45 years suffered from paraneoplastic forms, most commonly with ovarian teratomas [15, 86]. The symptoms usually develop in similar phases including psychotic/catatonic symptoms ([14, 15]; Fig. 1) or in case of relapses [44].

### Predominant and isolated autoimmune psychosis

In addition to the established main neuropsychiatric syndromes, milder Ab-associated autoimmune disorders with predominant or even isolated schizophreniform psychosis have been described in individual cases [23–25, 27–29, 44, 54, 56, 83]. For a subgroup of 23 out of 571 (4%) patients with anti-NMDA-R encephalitis, Kayser and colleagues described episodes with purely psychiatric presentations. Five patients developed an initial encephalitis with isolated psychotic symptoms (0.9%), and 18 patients (3.2%) had isolated psychiatric symptoms during a relapse [44]. In the meantime, cases with isolated anti-NMDA-R Ab detection in the serum and typical [^18^F]fluorodeoxyglucose positron emission tomography (FDG-PET) alterations were published [28]. In a case collection of 46 classic psychiatric Hashimoto encephalopathy cases, 12 patients suffered from acute psychosis (26.1%), and one patient met the criteria for schizophrenia (2.2%) [59]. Abs against intracellular antigens also may be associated with classical schizophreniform syndromes in rare individual cases [24, 60]. Tissue-based assays helped to detect new antineuronal Abs with neuropil pattern and yet unspecified target epitopes [27]. For such psychiatric manifestations of AE, the concept of AP was suggested and consensus criteria for possible, probable, and definite AP have recently been proposed for the first time ([67]; Table 2).

**Table 2:** The criteria of possible, probable, and definite autoimmune psychosis [67]

Possible autoimmune psychosis	Probable autoimmune psychosis	Definite autoimmune psychosis
Psychotic episode with abrupt onset (less than 3 months) with at least one of the following: 1. Tumor, 2. Movement disorder (catatonia/dyskinesia), 3. Adverse response to antipsychotics indicative of neuroleptic malignant syndrome, 4. Severe/disproportionate cognitive dysfunction, 5. Decreased level of consciousness, 6. New seizures 7. Significant autonomic dysfunction (pathological fluctuant blood pressure, temperature or heart rate)	Meeting the criteria for possible AP and At least one of the following: 1. CSF pleocytosis (> 5 per mm^3^) 2. Bilateral brain abnormalities on T2-weighted fluid-attenuated inversion recovery MRI highly restricted to the medial temporal lobes Or two of the following: 1. “Encephalopathic” EEG alterations (i.e., spikes, spike-wave activity, rhythmic slowing, focal changes or extreme delta brush) 2. CSF specific OCBs and/or increased IgG index 3. The presence of a serum anti-neuronal antibody detected by cell-based assay After exclusion of alternative causes	Meeting the criteria for probable AP and Evidence for IgG anti-neuronal antibodies in CSF.

*AP* autoimmune psychosis, *CSF* cerebrospinal fluid, *EEG* electroencephalography, *IgG* immunoglobulin G, *MRI* magnetic resonance imaging, *OCBs* oligoclonal bands

### Red flags that should lead to antibody diagnostics

**Fig. 2:**
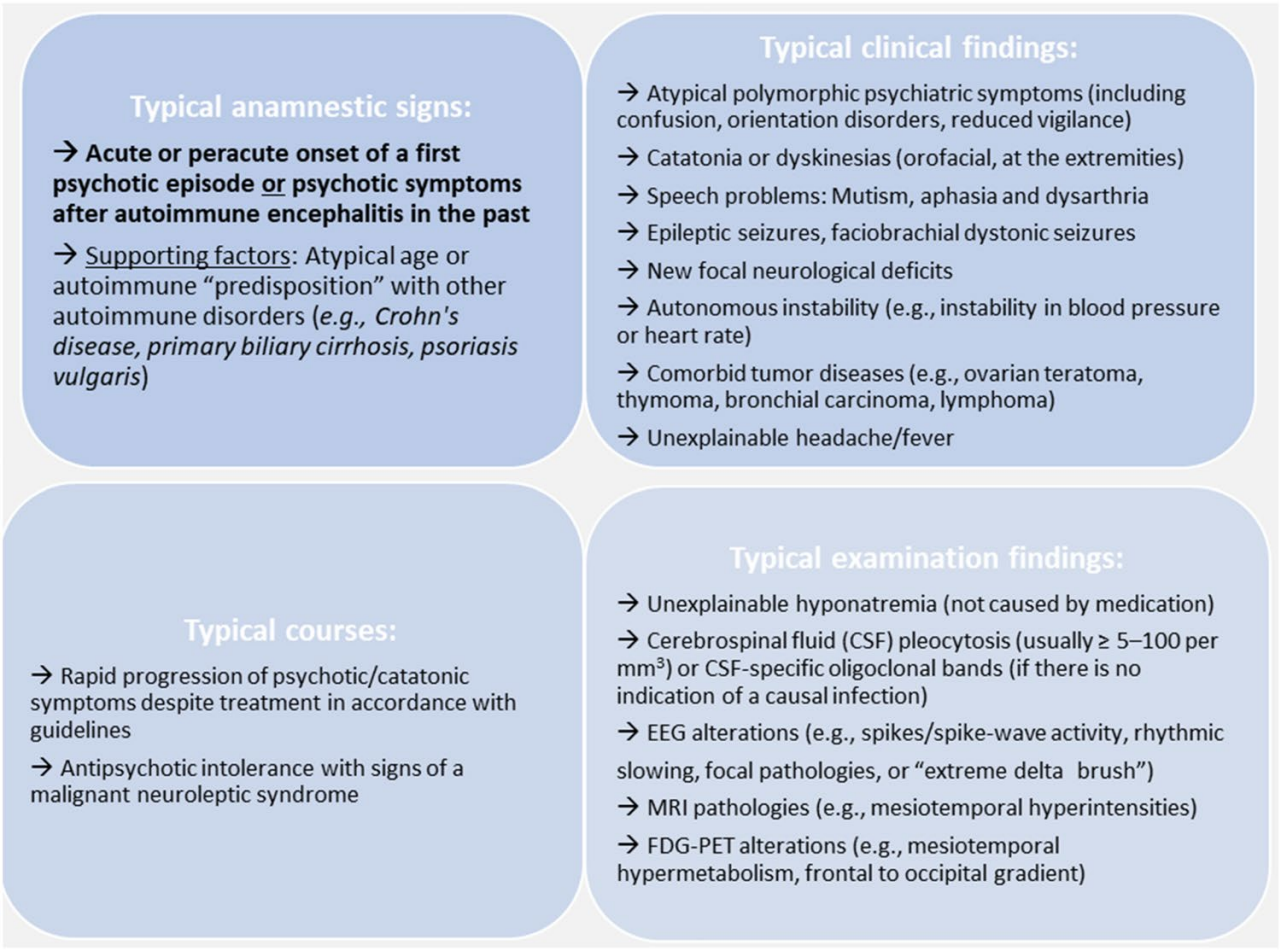
Red flags that should lead to antibody diagnostics (according to [2, 5, 22, 36, 53, 55, 61, 67, 74, 76, 77, 85]). *EEG* electroencephalography, *MRI* magnetic resonance imaging

The relatively rapid development of a psychotic syndrome, atypical and often polymorphic clinical symptoms and the presence of other neurological and/or medical symptoms are typical signs in autoimmune pathogenesis and thus should prompt broad Ab analyses [22]. Certain constellations in the course of the disease and typical additional findings should also trigger clinicians to consider the possibility of an AE/AP (Fig. 2; [2, 22, 36, 67, 76, 77, 84, 85]).

## Pathophysiology

### Established antineuronal antibodies

Abs against neuronal epitopes can be divided into Abs against cell surface antigens, which are most frequently associated with schizophreniform psychoses, and those against intracellular antigens [15, 36, 67].

- *Abs against cell surface antigens* These Abs bind to synaptic receptors, ion channels, or other cell surface proteins. This enables pathogenic Abs to lead to functional changes in electrophysiological signaling or synaptic transmission [8, 47, 68]. Therefore, they can have a direct pathogenic meaning. The exact pathophysiological processes are partly understood. Ab formation can be tumor-triggered. In addition, herpes simplex or other infections can act as triggers of the pathogenic process [4, 42]. Apart from that, Ab production can be the expression of autoimmune predisposition [15]. The initial hope that the anti-NMDA-R Abs at disease onset could provide an explanation for the glutamate hypothesis of schizophrenia [75, 87] could not be confirmed. Some of the largest Ab studies to date (with > 1000 schizophrenia patients), which were limited to blood serum examinations, have shown similar prevalence rates of different Abs (across all Ab classes, especially IgA and IgM isotypes) in the serum of patients with schizophrenia and controls, predominantly with very low Ab titers [13, 33]. At the same time, Ab detection in CSF appears to be less frequently [26, 64]; in a study of 124 patients with schizophrenia spectrum disorders, even all CSF tests were negative for antibodies against NMDAR, AMPAR, CASPR2, LGI1, and GABA_A/B_R [64].
- *Abs against intracellular antigens* Abs against non-synaptic intracellular antigens (e.g., Hu) typically occur paraneoplastically and have no direct pathogenic effect. They merely represent an epiphenomenon of a systemic tumor-triggered immune process. The cause of the inflammatory brain damage is a misguided response of cytotoxic T cells [51, 79]. There are often early and irreversible structural neuronal damages [79]. Abs against synaptic intracellular antigens are the “stiff-person spectrum” Abs against GAD65 and amphiphysin [51]. Anti-GAD65 Abs are more common idiopathically, and it has not been conclusively determined whether they have a pathogenetic significance or are only an epiphenomenon of another immune process [15].

### Systemic “possibly antineuronal” antibodies

These Abs do not bind exclusively to neuronal structures and can also be found together with antineuronal Abs in the context of an autoimmune predisposition [52]. Antinuclear antibodies (ANAs) can bind to ubiquitous nuclear structures (e.g., ds-DNA), but also to NMDA receptors and activate them. Therefore, excitotoxicity mediated by an acute NMDA receptor as well as subacute activation of microglia cells can lead to the destruction of synapses [62]. Thyroid Abs also occurs in about 13% of the healthy population [31], and serum Ab titers do not clearly correlate with symptom expression [48]; therefore, most authors have regarded them as an epiphenomenon [22].

## Diagnostic approach

### Indication for antibody analyses

The indication for serum and CSF Ab analyses results from the above-mentioned red flags (Fig. 2). The following considerations and operationalizations represent a kind of clinical consent among the authors who are all active in clinical diagnosis and management of new onset psychiatric and in particular psychotic patients. In the authors’ opinion, Ab measurements should be performed at least in the following constellation (compare with [36, 61, 67, 76, 77, 82, 85]):

**Table:** 

The combination of acute or peracute onset of a first schizophreniform psychotic episode *OR* psychotic symptoms after AE in the past *AND* according to Fig. 2: at least one typical clinical finding *OR* at least one sign of typical autoimmune course *OR* at least one typical examination finding [82, 85].	

**Fig. 3:**
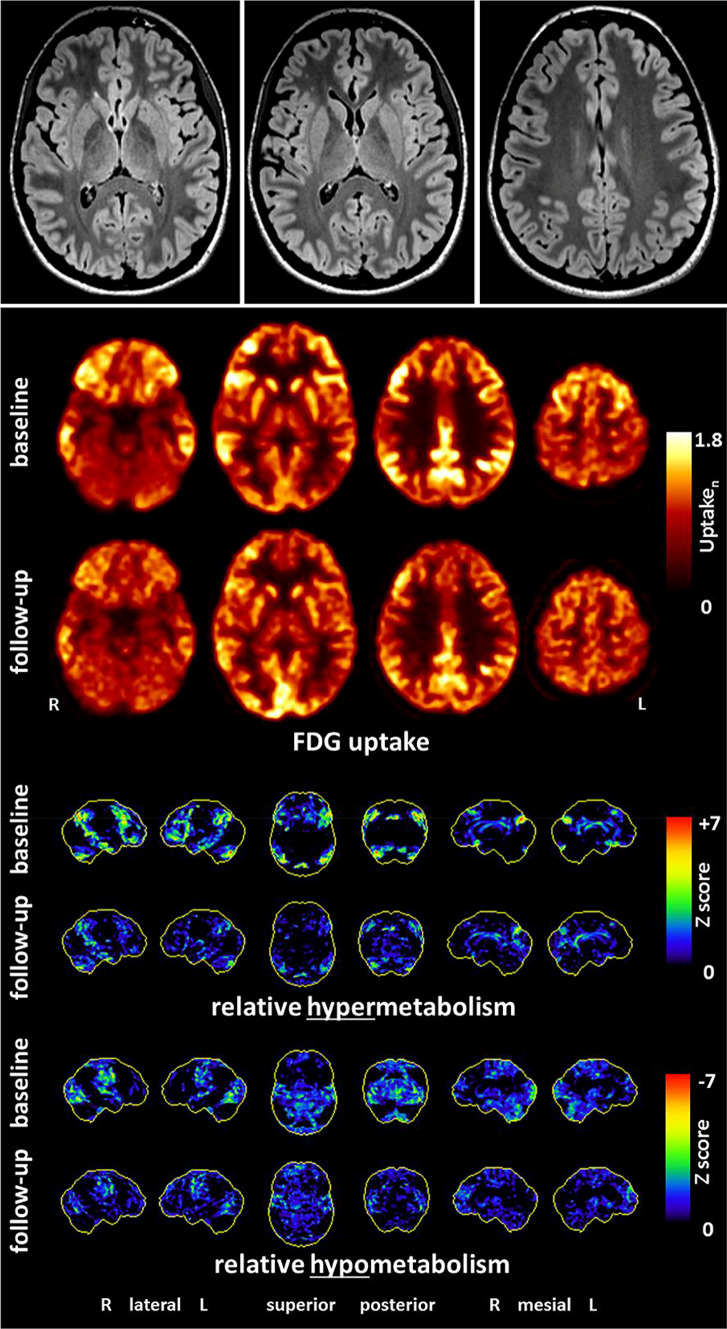
Findings of a 21-year-old female patient with probable anti-NMDA-R encephalitis. Magnetic resonance imaging depicted only a few slight, nonspecific bifrontal white matter lesions. [^18^F]fluorodeoxyglucose positron emission tomography showed pronounced relative hypermetabolism of her association cortices and a relative hypometabolism of the primary cortices (at baseline), which quickly improved during the follow-up examination after anti-inflammatory treatment (^©^Endres et al., 2019, Front Neurol. Nov 5 [28]: https://www.frontiersin.org/articles/10.3389/fneur.2019.01086/full)
- *Tumor screening* Tumor screening is essential in the event of the detection of paraneoplastic, onconeural antineuronal Abs.

All serum-Ab findings should be interpreted in the context of extended history data, the clinical syndrome, and the examination findings (especially including CSF Ab testing; [51, 82, 85]). The following investigations are suggested for patients with potential immunological genesis [82, 85]:

- *Extended history:* Infections/infectious prodroms and tumors should be looked for as possible triggers of Ab production. Attention should also be paid to a predisposition for immunological systemic diseases (presence of rheumatological diseases, inflammatory skin diseases, etc.). In addition, risk factors (such as earlier epileptic seizures, earlier episodes with encephalitides, infections), systemic signs (e.g., CNS or gastrointestinal symptoms), patient’s medication history (e.g., tolerability of antipsychotics), and family history should be inquired into.
- *Medical and neurological physical examination:* The medical examination should focus on possible signs for autonomic dysfunction or feverish conditions. In addition, attention should be paid to newly occurring neurological symptoms such as dyskinesia, or myoclonus.
- *Neuropsychological testing:* Neuropsychological testing should be considered to objectify more subtle cognitive deficits and to establish an objective follow-up parameter. The corresponding diagnostics can be based on standards of the established German GENERATE network (https://generate-net.de/generate-sops.html), which recommends carrying out bedside screening tests such as the Montreal Cognitive Assessment and extended tests such as the Test Battery for Attention Testing, Verbal Learning and Memory Test, Ray Rey-Osterrieth Complex Figure Test, or Frontal Assessment Battery, etc.
- *Laboratory measurements:* The basic parameters of CSF are very important for differential diagnostic considerations. Pleocytosis or CSF-specific OCBs provide information about a possible inflammatory process in the CNS. Based on the level of pleocytosis, autoimmune and infectious inflammations can often be distinguished [69]. Autoimmune genesis is usually accompanied by mild pleocytosis (from ≥ 5 to 100 per mm^3^; [31]), and the albumin quotient CSF/serum informs about the blood-CSF-barrier function, which should be assessed using the Reiber scheme [39, 72]. Serological analyses should exclude hyponatremia, which can be associated with anti-LGI1 Abs [88]. Box 2 puts forward a proposal for a two-step Ab diagnostic approach (compare with [82]). The determination of CSF is more sensitive for some Abs against established neuronal surface antigens; up to 14% of patients with anti-NMDA-R encephalitis had anti-NMDA-R Abs only in CSF [32]. The determination of Abs in serum and CSF enables the calculation of Ab indices (normalized to the total IgG ratio CSF/blood and the BBB function; [92]). Infectious (e.g., viral encephalitis), toxic, and other causes should be excluded.
- *EEG:* It is a sensitive, although not very specific, tool in the diagnosis of AEs [48, 86]. EEG examinations should, therefore, be carried out on a low-threshold basis [82, 85].
- *Imaging:* In LEs, MRI diagnostics usually show mesiotemporal hyperintensities in the T2 or FLAIR sequences [35]. In AEs with Abs against neuronal cell surface antigens, MRI often remains inconspicuous [35, 86]. The following sequences are suggested by the German GENERATE network: FLAIR axial + FLAIR coronary hippocampal view, T2 coronary, DWI axial and coronary, T2* axial or SWI, T1 + contrast agent axial, T1-MPRAGE (1 × 1 × 1 mm; before contrast agent; https://generate-net.de/generate-sops.html). If the findings remain unclear, an FDG-PET examination can be considered for specific questions. Compared to MRI, FDG-PET possibly has higher sensitivity for inflammatory changes ([5, 28, 35]; Fig. 3).

Box 2: Methodological aspects and suggestions for two-step antibody measurements **Basic measurement methods for the detection of antineuronal antibodies against cell surface antigens (especially against NMDA-R; [**41, 47, 76, 77, 82, 85**]):**

- *Screening tests:* Commercially available cell-based assays (CBAs) using indirect immunofluorescence (IF) on fixed cells expressing synaptic or neuronal cell surface proteins (also called “biochip assays”) are often used for screening. These tests might be less sensitive in patients with psychosis. However, they allow directly an exact detection of the target epitope.
- CBAs on live mammalian cells (so-called live CBAs) might show higher sensitivity for some surface antibodies (e.g., AMPA-R-abs); however, they are currently available only in special laboratories.
- *Tissue-based assays:* IF or immunohistochemical screening tests on brain sections of rodents can also detect previously unknown Abs. With their application, the percentage of “seronegative” cases is expected to decrease. Commercially available tissue-based tests are considered to be less sensitive than research laboratory approaches.

**Basic antibody screening for patients with schizophreniform psychoses should contain at least the most common IgG antibodies against the following antigens** [82**]:**

- NMDA-R, CASPR2, LGI1, AMPA-R, GABA_B_-R, GAD65 (in serum and CSF).
- Hu, Ri, Yo, CV2/CRMP5, Ma2 [Ta], Amphiphysin (in serum, CSF testing can be added if the serum is positive).
- TPO, TG, ANA (in serum).

**In the second step (in cases of negative screening and justified suspicion—compare** Table 3**), IgG antibodies against the following antigens can be added** [82**]:**

**Table 3:** The most important known autoantibodies that can be associated with symptoms of schizophreniform psychoses [9, 15, 31, 34, 36, 37, 38, 48, 63, 71, 79, 81, 82, 84, 85, 90]

Antigen	Established neuropsychiatric syndrome(s)	Typical symptomatology	Tumor association
Antibodies against neuronal cell surface antigens			
AMPA-R (GluR1/2)	Limbic encephalitis	Atypical psychosis, memory deficits, confusion	In approx. 65%, mostly with small cell bronchial carcinoma or thymomas
CASPR2	Morvan syndrome; limbic encephalitis	Psychotic and depressive symptoms, memory disorder, sleep disorder, neuromyotonia	In approx. 20–50% of patients (with morvan syndrome) thymomas
DPPX	Encephalitis, hyperekplexia, stiff-person spectrum	Delusion, hallucinations, cognitive deficits, confusion, diarrhea and other gastrointestinal symptoms, weight loss, hyperekplexia	Lymphoma in < 10%
GABA_A_-R	Limbic encephalitis with refractory epileptic seizures, epileptic status	Catatonia, therapy-refractory seizures, epileptic status	Tumors are unusual, thymomas in < 5%
GABA_B_-R	Limbic encephalitis with early and pronounced seizures	Memory deficits, seizures, orolingual dyskinesia	In approx. 50%, mostly with small cell bronchial carcinoma
Glycine-R	Progressive encephalomyelitis with rigidity and myoclonus (PERM), Stiff-Person syndrome	Psychotic symptoms, behavioral changes, rigidity, myoclonus	In < 5%, there is an association with thymoma, bronchial carcinoma and lymphomas
IgLON5	Encephalitis with sleep disturbance	Hallucinations, depressiveness, sleep apnea, NREM + REM sleep behavior disorder and brainstem dysfunction (dysphagia, ataxia)	No tumor association known
LGI1	Limbic encephalitis	Polymorph psychotic symptoms, depression, REM sleep disorders, memory deficits up to dementia, confusion, faciobrachial dystonic seizures, hyponatremia	In 5–10% thymomas
mGluR5	Limbic encephalitis	Behavioral changes, emotional instability, memory deficits, confusion	In approx. 70% associated with Hodgkin lymphoma
Neurexin-3-alpha	Encephalitis (compareable with anti-NMDA-R encephalitis)	Changes of behavior, agitation, prodromal symptoms (fever, headache, gastrointestinal symptoms), seizures, confusion, disturbed consciousness	No tumor association known
NMDA-R (GluN1)	Anti-NMDA-R encephalitis	Psychosis, catatonia, epileptic seizures, movement disorders, autonomous instability, impaired consciousness	Depending on age and sex, total tumor association in approx. 40%, mostly ovarian teratomas
Antibodies against synaptic intracellular antigens			
Amphiphysin	Stiff-Person syndrome, encephalomyelitis	Memory deficits, confusion, rigidity, spasms	In > 90%: breast cancer and small cell bronchial carcinomas
GAD65	Limbic encephalitis, Stiff-Person syndrome, epileptic seizures, cerebellar dysfunction	Psychotic syndromes, autism and ADHD symptoms (in atypical cases), bizarre movement disorders, muscle rigidity, spasms, seizures, ataxia	Isolated anti-GAD65 Abs are rarely paraneoplastic (otherwise in max. 25% thymomas, small-cell bronchial carcinoma)
Antibodies against onconeuronal, non-synaptic intracellular antigens			
Hu, Ri, Yo, CV2 (CRMP5), Ma2 (Ta), SOX1, Tr/DNER^a^	Limbic encephalitis, cerebellar degeneration among others	Mixed neuropsychiatric symptoms, behavioral changes, neuropathies, gait disorders, seizures	In most cases (> 95%) tumor-associated, mostly SCLC and other neuroectodermal tumors, e.g. Merckel-Cell-Ca; testicular tumors in Ma2, breast/ovary in Yo, Hodgkin in Tr/DNER
Antibodies against thyroid tissue			
TG/TPO	Hashimoto encephalopathy (SREAT)	Paranoia, hallucinations, depressiveness, memory problems, confusion, epileptic seizures, speech disorders, myoclonus	No tumor association known
Rheumatic antibodies			
ANAs (anti-dsDNA/anti-Sm/anti-rib. P/anti-nucleosome antibodies), etc.	Neuropsychiatric SLE, etc.	Confusional states, anxiety, cognitive dysfunction, mood disorders, psychosis, headaches, seizures, stroke-like episodes, etc.	No tumor association known

^a^Directed against an extracellular neuronal antigen (delta/notch-like epidermal growth factor-related receptor). For a full list of abbreviations, see appendix

- GABA_A_-R, DPPX, mGluR5, Neurexin-3-alpha, IgLON5, Glycin-R (in serum and CSF).
- Additional rheumatological examinations:
  - In the case of positive ANA screening: Abs against dsDNA/ENA-differentiation (specification for Sm,nucleosome, etc.; in serum).
  - ANCA (specification for MPO and PR3; in serum).
  - Antiphospholipid Abs (anti-β2-Glykoprotein-I Abs, anticardiolipin Abs, lupus anticoagulant; in serum/citrate tube).

**Immunofluorescence screening tests on brain sections of rodents (“tissue-based assays”) can also detect previously unknown antineuronal antibodies.**

For the second step, CSF material can be stored and cooled at 4 °C for at least four to 6 weeks; alternatively, the CSF material can be deep-frozen at − 80 °C [82].

*In line with the authors’ clinical experience, only the Abs most frequently associated with schizophreniform psychoses are mentioned.

### Organic differential diagnosis

Primary forms of schizophreniform psychoses must be distinguished not only from secondary Ab-mediated AEs but also from other CNS diseases (Table 4).

**Table 4:** The most important organic differential diagnoses [31, 45, 81, 82, 84, 85]

Inflammatory disorders	Non-inflammatory disorders
CNS infections (e.g., neuro-borreliosis, neuro-syphilis, Whipple’s disease, Herpes simplex virus encephalitis, HIV infection, Creutzfeldt-Jakob disease) Demyelinating CNS diseases (e.g., multiple sclerosis, acute disseminated encephalomyelitis); neuromyelitis optica-spectrum diseases Other rheumatological diseases with brain involvement (e.g., neurosarcoidosis, Behcet’s disease) Primary/secondary CNS vasculitis Other immunological diseases: Rasmussen encephalitis, CLIPPERS etc. Progressive multifocal leukoencephalopathy (JC-Virus infection)	Intoxication (illegal drugs such as amphetamines or cannabis) Inborn Errors of Metabolism (e.g., Niemann-Pick type C, acute intermittent porphyria, phenylketonuria, glycogen storage disorders) Mitochondriopathies Congenital disorders (e.g., velocardiofacial syndrome, agenesis of corpus callosum) Seizure disorders (e.g., temporal lobe epilepsy, paraepileptic psychoses) Endocrinological diseases (e.g., Cushing’s disease, hypoparathyroidism, hyperparathyroidism) Craniocerebral trauma Vitamin deficiency (e.g., B1, folic acid, B12) Toxic-metabolic causes (e.g., anticonvulsants, steroid treatment; hepatic/uremic encephalopathy) Vascular hypoxic damage (strategic stroke lesions) Neoplasias (e.g., gliomas, lymphomas, meningitis neoplastica) Basal ganglia diseases (e.g., Parkinson’s disease, chorea minor, Wilson’s disease, pantothenate-kinase associated neurodegeneration, Huntington’s disease) Neurodegenerative-dementia syndromes (e.g., frontotemporal dementia, Lewy body dementia etc.) Creutzfeldt-Jakob disease

## Therapeutic experiences and considerations

For the treatment of AE/AP, not only are the classical symptomatic therapy approaches available, but more causal therapy options also exist with immunosuppressive agents and in case of paraneoplastic disease with tumor treatment. Immunosuppressive and tumor therapy should be coordinated in a multidisciplinary setting [76, 77, 82]. Because controlled therapy studies are not yet available, immunosuppressive treatments have so far been carried out in the form of individual therapy trials [79, 90].

### Symptomatic treatment

The risk for extrapyramidal motor side effects seems to be increased in patients with AEs [49, 67, 76, 77]. Therefore, psychotic symptoms in the context of AP can be symptomatically treated with antipsychotics with a low risk for motor side effects [76, 77]. Benzodiazepines can be used for anxiolysis and sedation and, in higher doses, for the treatment of catatonic symptoms [76, 77].

### Causal immunosuppressive/tumor treatment

**Fig. 4:**
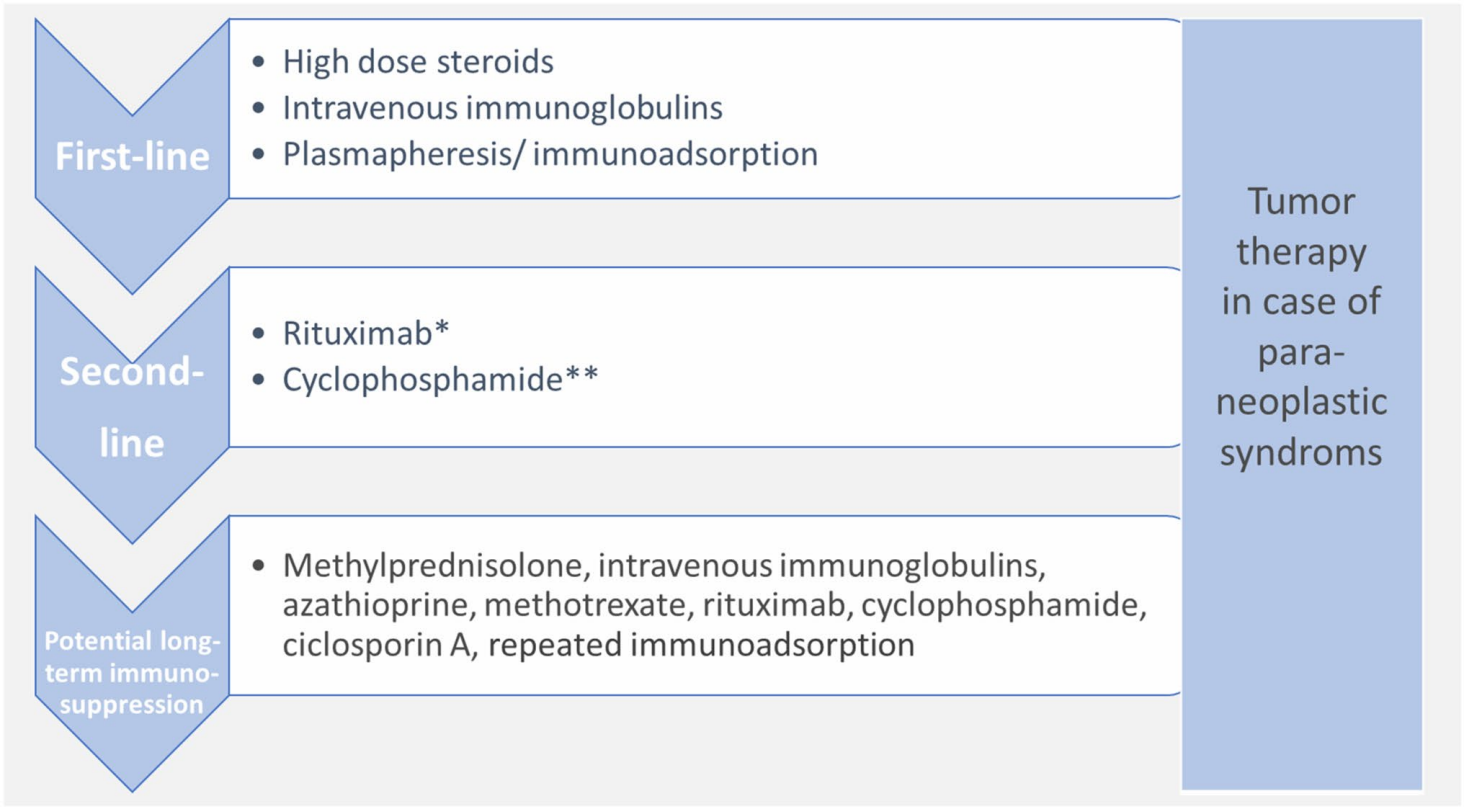
Therapeutic experiences and considerations for patients with autoimmune encephalitides and established antineuronal antibodies [11, 51, 58, 76, 77, 79, 82]. However, in individual cases, special features must be taken into account, depending on the individual autoantibodies/syndromes/circumstances. *Rituximab is increasingly used as a first-line therapy. **Treatment with cyclophosphamide should be used only with caution in young patients because of the relevant germ cell damage

The first-line therapy for established AEs is high-dose steroids (e.g., 500–1000 mg methylprednisolone over three to five days; [11, 76, 77, 82, 84]). Possible steroid-induced affective, suicidality, psychotic, and other side effects must be explained in advance [30] and closely monitored. Based on previous experiences, intravenous immunoglobulins or plasmapheresis/immunoadsorption can also be used as a first-line treatment [31, 51, 58, 76, 77, 79]. Rituximab or cyclophosphamide are recommended as “escalation”/“second line” therapies [11, 31, 51, 58, 76, 77, 79]. If relapse prevention turns out to be necessary, azathioprine, mycophenolate mofetil, or methotrexate are often used ([51]; Fig. 4). The decision for immunomodulatory maintenance/relapse prevention therapies is often complicated, depending on several factors, and should, therefore, only be made after a multidisciplinary discussion and under regular follow-up investigations. Depending on the Ab type, slightly different approaches have been established, which cannot be discussed in detail here. The aim of tumor treatment in paraneoplastic syndromes is to switch off the ectopic antigen source that maintains the autoimmune process ([79], Table 3).

### Limitations

The recommendations worked out here for Abs assessment and respective diagnostic and therapeutic consequences in schizophreniform psychoses were based on consensus from emerging clinical evidence rather than from systematic randomized studies as is the case with the present recommendations for diagnosis and treatment of AE [31]. Beyond, it should be recognized that indeed both well-established clinical terms (like encephalitis, encephalopathy, neuroinflammation) and newly proposed terms (such as AP, AE) are hardly exactly defined, thus for clinical use typically represent just clinical case definitions based on respective limited and steadily emerging clinical consensus [6, 12]. In addition, the possibility of underlying so far not identified new Abs is also limiting the whole issue. Finally, it should be pointed out again that low-positive serum antineuronal Ab titers without signs of brain involvement may occur nonspecifically and do not provide indication for treatment [13, 33, 50, 67].

## Conclusion

AE/AP represent a new field for psychiatry. The exact prevalence and thus clinical relevance of classical psychotic manifestations of AEs cannot yet be clearly established. However, the fact that predominant and even isolated psychotic clinical pictures may arise as a result of such AEs in certain cases is casuistically proven for most of the subtypes discussed here and already led to the first immunological treatment trials in Ab seropositive patients with psychosis [50]. Additionally, the topic has been captured in the new German S3 guideline for schizophrenia [20]. Future randomized-controlled and multimodal trials also taking into consideration CSF-results and Ab-titers are needed to shed more light on the relationship between the Abs and the outcome of psychosis discussed here.
